# HSF-DETR: A Special Vehicle Detection Algorithm Based on Hypergraph Spatial Features and Bipolar Attention

**DOI:** 10.3390/s25144381

**Published:** 2025-07-13

**Authors:** Kaipeng Wang, Guanglin He, Xinmin Li

**Affiliations:** Science and Technology on Electromechanical Dynamic Control Laboratory, Beijing Institute of Technology, Beijing 100081, China; 3120215105@bit.edu.cn (K.W.); 3120225110@bit.edu.cn (X.L.)

**Keywords:** special vehicle detection, RT-DETR, multi-scale feature fusion, deep learning, object detection

## Abstract

Special vehicle detection in intelligent surveillance, emergency rescue, and reconnaissance faces significant challenges in accuracy and robustness under complex environments, necessitating advanced detection algorithms for critical applications. This paper proposes HSF-DETR (Hypergraph Spatial Feature DETR), integrating four innovative modules: a Cascaded Spatial Feature Network (CSFNet) backbone with Cross-Efficient Convolutional Gating (CECG) for enhanced long-range detection through hybrid state-space modeling; a Hypergraph-Enhanced Spatial Feature Modulation (HyperSFM) network utilizing hypergraph structures for high-order feature correlations and adaptive multi-scale fusion; a Dual-Domain Feature Encoder (DDFE) combining Bipolar Efficient Attention (BEA) and Frequency-Enhanced Feed-Forward Network (FEFFN) for precise feature weight allocation; and a Spatial-Channel Fusion Upsampling Block (SCFUB) improving feature fidelity through depth-wise separable convolution and channel shift mixing. Experiments conducted on a self-built special vehicle dataset containing 2388 images demonstrate that HSF-DETR achieves mAP50 and mAP50-95 of 96.6% and 70.6%, respectively, representing improvements of 3.1% and 4.6% over baseline RT-DETR while maintaining computational efficiency at 59.7 GFLOPs and 18.07 M parameters. Cross-domain validation on VisDrone2019 and BDD100K datasets confirms the method’s generalization capability and robustness across diverse scenarios, establishing HSF-DETR as an effective solution for special vehicle detection in complex environments.

## 1. Introduction

Special vehicle target detection, as an important research direction in computer vision, has extensive application prospects and significant practical value in traffic monitoring, industrial inspection, reconnaissance, disaster rescue, and other domains [1,2]. Unlike ordinary vehicles, special vehicles typically possess complex structural features, diverse appearance forms, and specific functional components, making their detection and recognition face greater challenges [3]. Accurate and efficient special vehicle detection systems can not only improve traffic management efficiency and enhance industrial safety monitoring capabilities but also provide critical technical support for emergency rescue and equipment maintenance [4].

In this paper, special vehicles refer to specialized military and civilian vehicles including but not limited to main battle tanks, armored personnel carriers, self-propelled artillery, mobile radar systems, fire trucks, ambulances, and engineering vehicles. These vehicles are characterized by unique structural features such as armored hulls, rotating turrets, specialized equipment mountings, and distinctive geometric configurations that differentiate them from conventional civilian vehicles. The detection of such vehicles presents unique challenges including complex geometric structures, camouflage patterns, partial occlusion by terrain features, varying scales in aerial imagery, and the need to identify specific components like turrets and sensor arrays that are crucial for vehicle classification and threat assessment.

Traditional special vehicle target detection methods primarily rely on hand-crafted feature extractors and classifiers, such as the Histogram of Oriented Gradients (HOG), Scale-Invariant Feature Transform (SIFT), and Support Vector Machines (SVMs) [5]. Dalal and Triggs [6] proposed a vehicle detection method based on the HOG and SVMs, improving detection accuracy by constructing multi-scale feature pyramids. Zheng et al. [7] combined geometric and texture features of special vehicles to design a vehicle detection algorithm for high-resolution remote sensing images.

With the rapid development of Convolutional Neural Networks (CNNs), special vehicle target detection methods based on deep learning have achieved significant progress. With the rapid development of Convolutional Neural Networks (CNNs), special vehicle target detection methods based on deep learning have achieved significant progress [8]. Wang et al. [9] proposed a multi-scale feature fusion method based on deep residual networks, significantly improving vehicle detection accuracy. Liu et al. [10] designed an attention-enhanced feature extraction network, effectively improving special vehicle detection performance under complex backgrounds. Chen et al. [11] proposed a domain adaptation method for multi-view special vehicle detection, addressing cross-scene detection challenges. Ma and Xue [5] conducted a comprehensive review of deep learning-based vehicle detection methods, highlighting the widespread application of two-stage and single-stage detectors in intelligent transportation systems. With the introduction of self-attention mechanisms and Transformer architectures, detection performance has been further enhanced.

In recent years, general object detection frameworks have demonstrated good performance and strong practicality in the special vehicle detection domain. These methods are typically categorized into two types: single-stage detectors (such as YOLO series [12,13], SSD [14]) and two-stage detectors (such as Faster R-CNN [15], Mask R-CNN [16]). Single-stage detectors directly predict target locations and categories with high inference speed; two-stage detectors first generate candidate regions and then perform classification and refinement, typically achieving higher detection accuracy. The YOLO series, with its excellent speed-accuracy balance, has been widely adopted in real-time special vehicle detection applications. Zaidi et al. [17] reviewed recent deep learning object detection models, analyzing the application effects of various models in different scenarios. Improved versions such as YOLOv5 [18] and YOLOv7 [19] further enhanced feature extraction and multi-scale target detection capabilities.

Recently, Transformer-based detectors have become research hotspots due to their powerful global modeling capabilities. Carion et al. [20] proposed DETR, which first successfully applied Transformers to object detection, eliminating hand-crafted components in traditional detectors (such as anchors and non-maximum suppression), but with slow inference speed. To address this issue, Zhao et al. [21] proposed RT-DETR (Real-Time Detection Transformer), achieving efficient real-time inference through hybrid encoders and lightweight decoders. RT-DETR employs efficient hybrid encoders to process multi-scale features, achieving significant inference speed improvements while maintaining high accuracy through decoupled intra-scale interaction and cross-scale fusion [22].

Zhang et al. [23] proposed DINO (DETR with improved denoising anchor boxes), significantly improving DETR model performance and efficiency through contrastive denoising training, hybrid query selection methods for query initialization, and look-forward-twice box prediction schemes. Li et al. [24] explored vehicle logo recognition methods based on Swin Transformers, utilizing their efficient computation and global feature modeling capabilities to enhance key feature extraction for special vehicles. Wang et al. [25] designed a dynamic graph learning method based on content-guided spatial-frequency relationship reasoning. Although applied to DeepFake detection, its spatial-frequency hybrid attention mechanism provides new insights for enhancing feature expression capabilities, which can be transferred to vehicle key component recognition tasks. While recent works like Hyper-YOLO [26] have explored ε-ball semantic space hyperedges for general object detection, our approach specifically designs geometry-driven hypergraph structures tailored for special vehicle detection challenges. Unlike ε-ball semantic hyperedges that rely on feature similarity in embedding space, our geometric neighborhood-based hyperedges capture spatial relationships and structural patterns specific to armored vehicles and their components, enabling the more precise detection of rigid geometric structures such as turrets.

Despite significant progress in target detection technology in the special vehicle recognition domain, the following key challenges remain: insufficient feature extraction, where existing backbone networks struggle to capture subtle differences between targets and backgrounds and hierarchical relationships between components when processing high-resolution special vehicle images; inadequate multi-scale feature fusion, where traditional fusion networks fail to establish effective correlations between different scale features, leading to low detection accuracy for small components and difficulty distinguishing similar structures; unbalanced attention allocation, where existing encoders have uneven attention allocation for targets under complex backgrounds, limited long-range dependency modeling capabilities, and insufficient frequency-domain information processing, affecting model robustness; information loss during upsampling, where traditional upsampling methods suffer from feature accuracy loss, limited detail recovery capabilities, and insufficient inter-channel information interaction, resulting in blurred boundaries and inaccurate fine structure recognition.

Addressing the above challenges, this paper proposes HSF-DETR (Hypergraph Spatial Feature DETR), a special vehicle detection algorithm based on hypergraph spatial features and bipolar attention. The main contributions are as follows:

- A Cascaded Spatial Feature Network (CSFNet) backbone is proposed, based on Cross-Efficient Convolutional Gating (CECG) feature extraction modules. By combining hybrid state-space modeling with convolutional gating mixing units, it enhances the network’s detection capability for long-range special vehicles and their key components while improving model robustness under adverse conditions such as occlusion, lighting variations, and complex backgrounds.
- A Hypergraph-enhanced Spatial Feature Modulation (HyperSFM) feature fusion network is designed. This network models high-order feature correlations through hypergraph structures and combines Spatial Feature Modulation (SFM) to achieve efficient fusion and adaptive modulation between different scale features, significantly improving the system’s detection capability for vehicle key components.
- A Dual-Domain Feature Encoder (DDFE) is proposed, combining Bipolar Efficient Attention (BEA) and a Frequency-Enhanced Feed-Forward Network (FEFFN). Through innovative bipolar representation and frequency-domain enhancement mechanisms, it achieves more precise feature weight allocation and richer detail feature extraction, improving system detection accuracy and robustness under complex environments.
- A Spatial-Channel Fusion Upsampling Block (SCFUB) is developed. This module combines depth-wise separable convolution with channel shift mixing techniques, significantly enhancing feature fidelity and spatial consistency during upsampling while maintaining computational efficiency, effectively addressing fine recognition issues of special vehicle key components.

By integrating the above four innovative modules, HSF-DETR forms an end-to-end detection framework, achieving a balance between accuracy and efficiency in special vehicle detection tasks.

## 2. Related Work

### 2.1. Dataset Construction and Annotation Strategy

Special vehicle detection is a key technology in modern intelligent surveillance, emergency rescue, and reconnaissance domains. However, existing public datasets mainly focus on conventional civilian vehicles, lacking specialized datasets for special vehicles and their key components under complex environments, severely constraining the performance optimization of related detection algorithms in special scenarios. To address this issue, this research constructs a high-quality complex environment special vehicle detection dataset containing 2388 meticulously annotated images, divided into a training set (1671 images), validation set (239 images), and test set (478 images) at a 7:1:2 ratio. All images are precisely annotated by professional equipment identification experts to ensure data quality and annotation accuracy.

**Figure 1:**
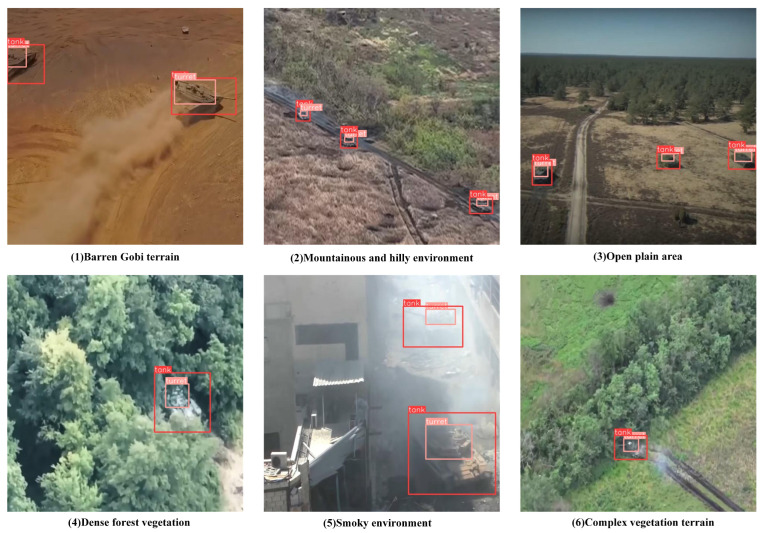
Representative samples from the self-built special vehicle detection dataset covering various challenging scenarios including barren desert terrain, mountainous environments, open plains, dense vegetation, smoke interference, and complex vegetation terrain.

As shown in Figure 1, the dataset covers various typical complex environment scenarios: (a) multi-target detection scenes in barren desert terrain; (b) concealed target recognition in mountainous and hilly environments; (c) long-range surveillance scenes in open plain areas; (d) occluded target detection under dense forest vegetation coverage; (e) low-visibility detection under smoke interference conditions; (f) camouflaged target recognition in complex vegetation terrain. Data collection primarily employs unmanned aerial vehicle bird’s-eye view imaging, supplemented by ground-level perspectives, comprehensively simulating diverse visual challenges in real-world applications. The dataset defines two core detection categories, tank (vehicle body) and turret (turret component), where the turret serves as a key identification feature of special vehicles. Its precise detection is crucial for vehicle type discrimination and intelligent decision making, providing important data support for algorithm research and engineering applications in related domains.

### 2.2. RT-DETR Baseline Framework Model and Comparative Analysis

RT-DETR (Real-Time Detection Transformer) is an end-to-end real-time object detection model proposed by Baidu [21]. It mainly consists of three core components: backbone network, encoder, and decoder.

The backbone network includes ResNet and HGNetv2 series. Unlike traditional DETR, RT-DETR extracts multi-scale features from the last three stages (S3, S4, S5) of the backbone network, providing rich multi-scale information for subsequent encoders.

The encoder consists of two key modules: the Attention-based Intra-scale Feature Interaction (AIFI) module and the CNN-based Cross-scale Feature Fusion (CCFF) module. The AIFI module utilizes self-attention mechanisms to process feature interactions within the same scale, enhancing feature expression capabilities, while the CCFF module is responsible for fusion between different scale features, comprehensively utilizing multi-scale information.

The decoder adopts a Transformer structure with auxiliary prediction heads. Based on encoder outputs, RT-DETR introduces an innovative IoU-aware Query Selection mechanism for selecting high-quality initial target queries. The decoder iteratively optimizes these target queries to generate final bounding boxes and confidence scores.

RT-DETR (Real-Time Detection Transformer) demonstrates superior performance compared to the YOLO series in real-time scenarios due to its efficient hybrid encoder design and end-to-end detection paradigm without post-processing requirements like NMS. RT-DETR-R50 achieves 53.1% AP at 108 FPS on the COCO dataset, outperforming YOLOv8 in both speed and accuracy while eliminating inference delays caused by non-maximum suppression.

Recent advances have also explored hypergraph structures for object detection. The Hyper-YOLO series [26] introduces hypergraph computation-empowered semantic collecting and scattering frameworks, demonstrating the potential of hypergraph structures in capturing complex feature relationships. These methods utilize ε-ball semantic space hyperedges to model high-order dependencies between features through semantic similarity in embedding space.

### 2.3. Feature Fusion Networks

The Feature Pyramid Network (FPN) is a key technology in object detection, aimed at addressing multi-scale object detection problems [27]. Since Lin et al. proposed the FPN in 2017, this technology has become a standard component of many object detectors [28]. In recent years, researchers have proposed various improved FPN variants to enhance feature representation capabilities.

Ghiasi et al. proposed the NAS-FPN, discovering better feature pyramid structures through neural architecture search [29]. To better handle small target detection, Deng et al. proposed the Extended Feature Pyramid Network, enhancing the FPN’s sensitivity to small-scale targets [30]. Addressing information loss during feature fusion, Zhu et al. proposed an improved Feature Pyramid Network (ImFPN), which includes segmentation attention modules and similarity-based fusion modules, better adapting to instances of different scales [31]. Additionally, some researchers have explored multi-path attention mechanisms to enhance FPN’s representation capabilities, such as MAFPNs (Multi-scale Attention-based Feature Pyramid Networks), which can simultaneously consider scale, spatial, and channel information, more comprehensively processing multi-scale inputs [32].

The research progress of these Feature Pyramid Networks provides a theoretical foundation for the proposed hypergraph spatial features and bipolar attention mechanisms in this paper. We will build upon these cutting-edge works, combined with the special requirements of special vehicle detection, to propose more effective feature extraction and fusion strategies.

## 3. Method

**Figure 2:**
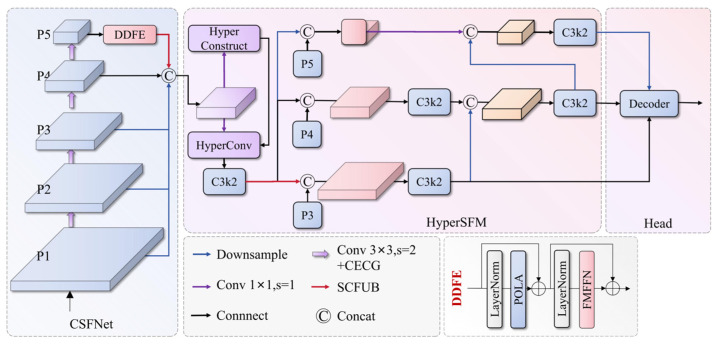
Overall architecture of the proposed HSF-DETR framework showing the integration of CSFNet backbone with CECG modules, HyperSFM feature fusion with Hypergraph Relational Aggregator, and Spatial Feature Modulation, DDFE combining Bipolar Efficient Attention and Frequency-Enhanced Feed-Forward Network, and SCFUB upsampling modules with cross-spatial channel mixer.

This paper proposes HSF-DETR, consisting of four key modules. First, the Cascaded Spatial Feature Network (CSFNet) extracts multi-scale features, followed by the Dual-Domain Feature Encoder (DDFE) combining bipolar directional attention and frequency-domain modulation encoders. Input to the Hypergraph-enhanced Spatial Feature Modulation (HyperSFM) feature fusion network utilizes hypergraph structures to model high-order feature correlations. The Spatial-Channel Fusion Upsampling Block (SCFUB) enhances upsampling quality through channel shift mixing. These four modules work collaboratively to form an end-to-end detection framework, significantly improving detection accuracy and robustness for special vehicles and their key components under complex environments while maintaining high computational efficiency. The overall framework of HSF-DETR is shown in Figure 2. The following subsections will detail each component module of HSF-DETR and its working principles.

### 3.1. Cascaded Spatial Feature Network (CSFNet)

**Figure 3:**
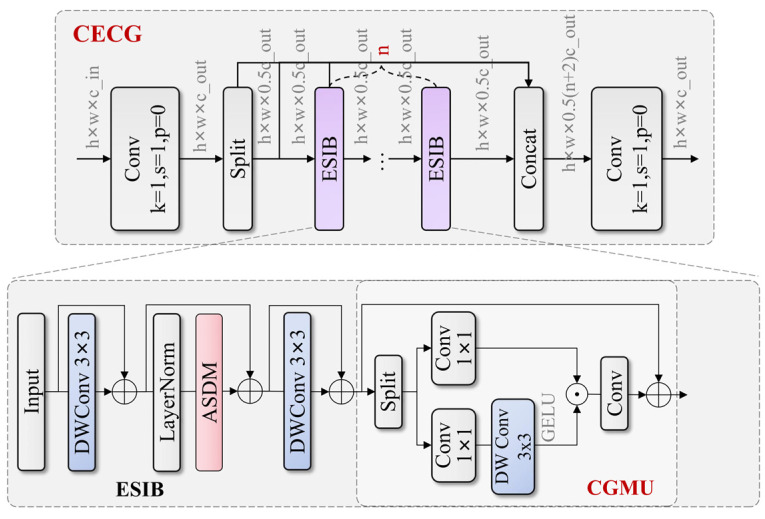
Structure of the CECG module featuring cascaded ESIB with Adaptive State Decomposition Modules and CGMU with depth-wise convolution and element-wise multiplication operations.

Traditional ResNet backbone networks face issues of insufficient feature extraction, limited representation capabilities, and parameter redundancy when processing high-resolution special vehicle images. Particularly when recognizing large armored vehicles and their rotating observation chambers and other key components in complex terrain, networks struggle to capture subtle differences between targets and backgrounds and hierarchical relationships between components. To address these problems, we propose a Cascaded Spatial Feature Network (CSFNet) backbone based on Cross-Efficient Convolutional Gating (CECG) modules, with the overall structure of CECG shown in Figure 3. This network enhances the network’s detection capability for long-range special vehicles and their key components by combining hybrid state-space modeling with convolutional gating linear units while improving model robustness under adverse conditions such as occlusion, lighting variations, and complex backgrounds. CSFNet demonstrates significant advantages in application scenarios such as industrial equipment monitoring, traffic management, and disaster rescue vehicle identification.

Our hypergraph topology was dynamically constructed based on geometric neighborhood relationships rather than being fixed or purely learned. Specifically, hyperedges are defined by grouping 3–8 feature nodes within a local spatial window of size 7 × 7. The connectivity criterion was based on spatial distance threshold τ combined with feature similarity. For each potential hyperedge, we computed the geometric centroid of candidate nodes and included nodes that satisfy both spatial proximity (distance < τ × receptive_field_size) and feature similarity (cosine similarity > 0.6). The threshold τ was empirically set to 0.4 based on validation experiments. Each hyperedge typically connects 4–6 nodes representing spatially coherent feature regions, enabling the capture of local geometric patterns while maintaining computational efficiency.

The CECG module achieves feature enhancement through improved Cross-Stage Partial [33] connections and efficient visual hybrid state-space modeling. The overall mathematical expression of this module can be formalized as follows:

(1) $$Y=C_{2}\left(C_{1}{\left(X\right)}_{1},C_{1}{\left(X\right)}_{2},M_{1}\left(C_{1}{\left(X\right)}_{2}\right),\cdots ,M_{n}\left(C_{1}{\left(X\right)}_{2}\right)\right)$$

where $C_{1}$ and $C_{2}$ represent two convolutional layers, respectively, $M_{i}$ represents the i-th Efficient Spatial Interaction Block (ESIB) module, and $\left[\cdot \right]$ represents feature concatenation operation. The workflow first maps input features X to hidden space through $C_{1}$ and divides them into two parts, where one part is directly transmitted and the other part is processed through cascaded ESIB modules, finally fusing all features through $C_{2}$.

The ESIB module adopts residual adaptive learning mechanisms and convolutional gating linear units, effectively improving the network’s representation capabilities. Its mathematical expression is as follows:

(2) $$X_{out}=X+\alpha_{3}\cdot F_{CG}\left(X+\alpha_{2}\cdot F_{ASDM}\left(X+\alpha_{1}\cdot F_{DW1}\left(X\right)\right)\right)$$

where $\alpha_{i}$ are learnable adaptive parameters, $F_{DW1}$ and $F_{DW2}$ are depth-wise separable convolutions, $F_{ASDM}$ is the Adaptive State Decomposition Module (ASDM), and $F_{CG}$ is a feed-forward network based on Convolutional Gating Mixing Unit (CGMU). The computation process of CGMU can be expressed as follows:

(3) $$F_{CG}\left(X\right)=X+W_{2}\left(DW\left(X_{1}\right)⊙X_{2}\right)$$

where $X_{1}$ and $X_{2}$ are two parts of input X after projection through $W_{1}$, DW represents depth convolution, and ⊙ represents element-wise multiplication. This design enables the module to more effectively process vehicle contour and key component detail features, improving model recognition capabilities under different viewing angles and partial occlusion conditions.

Gating mechanism was chosen over traditional activation functions like ReLU or SE-blocks for several specific reasons relevant to special vehicle detection. Unlike ReLU, which applies element-wise thresholding, gating enables selective information flow based on learned importance weights, which is crucial for distinguishing between vehicle components and background clutter. Compared to SE blocks that focus on channel-wise attention, our convolutional gating mechanism preserves spatial relationships essential for geometric structure recognition in armored vehicles. The multiplicative gating operation ($X_{1}⊙X_{2}$) allows fine-grained control over feature activation based on local context, enabling the model to adaptively emphasize vehicle contours, turret boundaries, and other distinctive geometric features while suppressing irrelevant background information.

**Figure 4:**
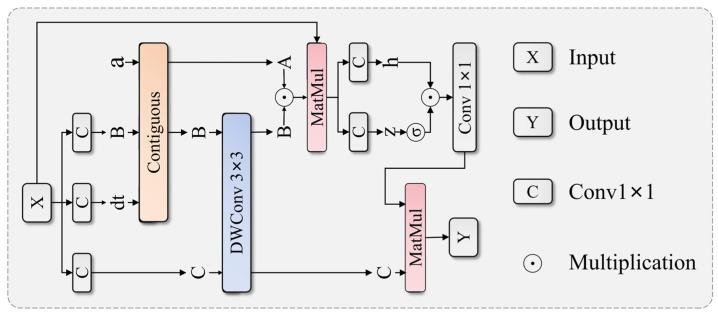
Architecture of the Adaptive State Decomposition Module (ASDM) showing input projection to B, C, dt parameters, continuous convolution processing, MatMul operations for state interaction, and output generation through selective state decomposition and hybrid state interaction mechanisms.

The ASDM is key for the network to capture long-range dependency relationships, with its structure shown in Figure 4. It first decomposes input features into state parameters through projection:

(4) $$\left[B,C,dt\right]=DW\left(W_{proj}\left(X\right)\right)$$

Then, the computation process for state interaction and feature enhancement is as follows:

(5) $$A=softmax\left(dt+A_{0}\right),h_{1}=W_{hz}\left(A\right)⊙B$$

(6) $$Y=h_{1}⊙\sigma \left(z\right)\cdot C$$

where $A_{0}$ is a learnable initial state parameter, $W_{proj}$ and $W_{hz}$ are projection transformations, and σ is an activation function. This module effectively captures spatial relationships and structural features between various components of armored vehicles through selective state decomposition and hybrid state interaction.

Based on the CECG feature extraction module, the CSFNet backbone network has achieved significant success in recognition tasks for special vehicles and their key components. Compared to traditional backbone networks, CSFNet demonstrates stronger feature expression capabilities and environmental adaptability, accurately recognizing large armored vehicles and their functional components under challenging conditions such as complex backgrounds, lighting variations, and partial occlusion. This network optimizes the balance between computational efficiency and accuracy, ensuring both real-time performance and detection quality.

### 3.2. Hypergraph-Enhanced Spatial Feature Modulation (HyperSFM)

**Figure 5:**
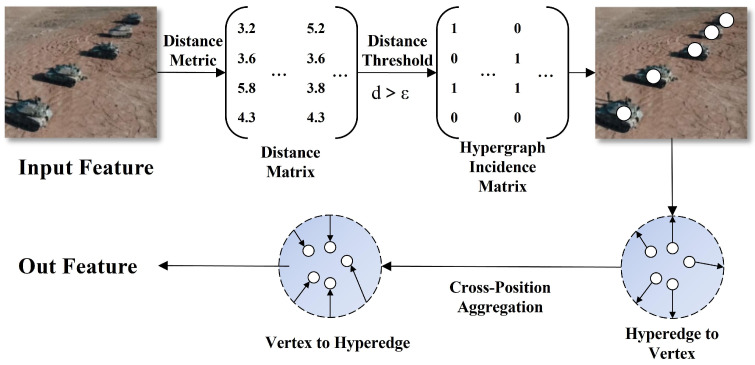
Hypergraph Relational Aggregator (HRA) demonstrating cross-position aggregation through hypergraph convolution operations for modeling high-order feature correlations.

Traditional feature fusion networks like CCFM (Conventional Cross-Feature Fusion Mechanism) face issues of limited feature expression capabilities, insufficient semantic information transmission, and poor scale variation adaptability when processing multi-scale special vehicle images. Traditional fusion methods struggle to establish effective correlations between different scale features, leading to low detection accuracy for small components, difficulty distinguishing similar structures, and high false detection rates under complex backgrounds. Addressing these challenges, we propose the Hypergraph-enhanced Spatial Feature Modulation (HyperSFM) feature fusion network, with its structure shown in Figure 5. This network achieves high-order correlation modeling and adaptive feature fusion between features through hypergraph structures and multi-scale feature modulation mechanisms, significantly improving system performance in applications such as traffic monitoring, industrial equipment monitoring, and rescue vehicle identification, particularly the detection capability for vehicle observation systems and other fine components.

HyperSFM combines hypergraph theory with feature modulation mechanisms. It collects hierarchical features through the semantic collecting module; then, it employs Hypergraph Relational Aggregator (HRA) to establish high-order dependency relationships between feature points, surpassing the limitation of traditional graph structures where edges can only connect two nodes; finally, it designs Spatial Feature Modulation (SFM) to achieve adaptive feature fusion.

HRA primarily establishes high-order correlations between feature points through hypergraph convolution operations, with its structure shown in Figure 5. Our hypergraph construction differs from existing semantic-based approaches by utilizing geometric neighborhood criteria. We define hyperedges based on spatial proximity and geometric relationships between feature points, which is particularly beneficial for capturing the rigid geometric structures and spatial arrangements of special vehicle components such as turrets.

Our hypergraph topology was dynamically constructed based on geometric neighborhood relationships rather than being fixed or purely learned. Specifically, hyperedges were defined by grouping 3–8 feature nodes within a local spatial window of size 7 × 7. The connectivity criterion was based on spatial distance threshold τ combined with feature similarity. For each potential hyperedge, we computed the geometric centroid of candidate nodes and included nodes that satisfy both spatial proximity and feature similarity. The threshold τ was empirically set to 4 based on validation experiments. Each hyperedge typically connects 4–6 nodes representing spatially coherent feature regions, enabling the capture of local geometric patterns while maintaining computational efficiency.

Its workflow first constructed feature hypergraphs and then performed bidirectional message passing and updates based on the hypergraph. The overall mathematical expression is as follows:

(7) $$X_{out}=\sigma \left(BN\left(H\left(X,G\left(X,\tau \right)\right)+X\right)\right)$$

where $X\in R^{B\times H\times W\times C}$ represents the reshaped feature matrix, $G\left(X,\tau \right)$ represents the hypergraph adjacency tensor constructed based on threshold τ, H represents hypergraph convolution operations, BN is batch normalization, and σ is an activation function. The mathematical expression for hypergraph convolution H is as follows:

(8) $$H\left(X,G\right)=M_{e2v}\left(M_{v2e}\left(W\left(X\right),G\right),G\right)$$

where W is a learnable linear transformation, $M_{v2e}$ and $M_{e2v}$ represent message aggregation operations from nodes to hyperedges and from hyperedges to nodes, respectively. This module can capture complex spatial relationships between key structural components of special vehicles through hyperedge connections of multiple nodes, providing powerful high-order feature representations for subsequent precise positioning and component recognition, effectively addressing challenges such as partial occlusion and complex backgrounds.

Modeling higher-order relationships through hypergraph structures enables generalization across diverse scene types by capturing complex spatial interdependencies that extend beyond pairwise relationships. In urban environments, hyperedges can simultaneously connect road surfaces, building facades, and vehicle positions to understand contextual relationships. For rural or battlefield scenarios, the same hypergraph mechanism captures relationships between terrain features, vegetation patterns, and vehicle camouflage effectiveness. This approach generalizes across different lighting conditions by modeling illumination-invariant geometric relationships rather than relying solely on appearance features.

**Figure 6:**
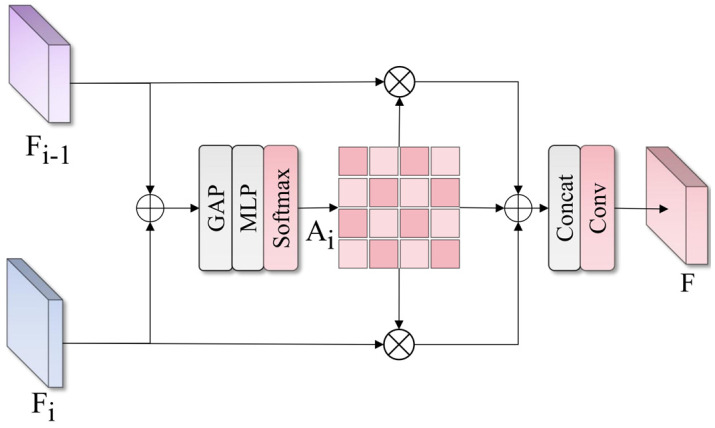
Spatial Feature Modulation (SFM) module implementing adaptive feature fusion through attention-weighted combination of multi-scale features.

The SFM module’s structure is shown in Figure 6. Its working principle is to adaptively adjust the contribution weights of each scale feature based on feature importance, with the overall mathematical expression as follows:

(9) $$O=\sum_{i=1}^{h}F_{i}⊙A_{i}$$

where $F_{i}$ represents the i-th input feature (after scale alignment), $A_{i}$ represents the corresponding attention weight, h is the number of input features, and ⊙ represents element-wise multiplication. The computation process for attention weights is as follows:

(10) $$A=Softmax{(W}_{2}(\sigma (W_{1}(GAP*(\sum_{i=1}^{h}F_{i})))))$$

where GAP represents global average pooling operation, $W_{1}$ and $W_{2}$ are MLP layer weights, and σ is a nonlinear activation function. SFM achieves more refined and adaptive feature fusion by learning the importance of different scale features, dynamically adjusting weights of each scale feature based on specific content of input images, effectively improving system detection accuracy for different sizes and states of special vehicle components, enabling the model to achieve high-performance recognition in complex monitoring and detection scenarios.

The HyperSFM feature fusion network successfully addresses multi-scale feature fusion challenges in special vehicle and key component detection through hierarchical perception, hypergraph modeling, and adaptive fusion design concepts. Compared to traditional methods, this network achieves richer feature correlation modeling through hypergraph structures, effectively capturing complex dependency relationships between various vehicle components. The overall network architecture optimization design enables the system to maintain high accuracy while preserving computational efficiency.

### 3.3. Dual-Domain Feature Encoder (DDFE)

**Figure 7:**
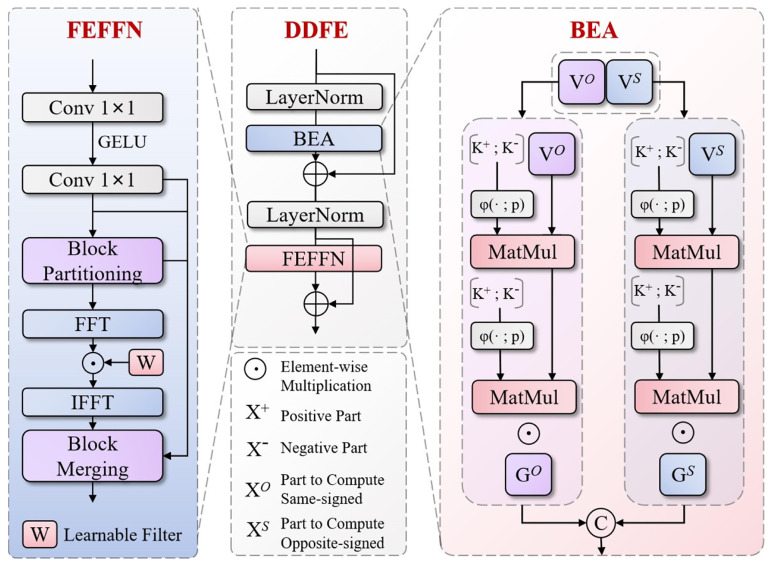
Dual-Domain Feature Encoder (DDFE) architecture combining Bipolar Efficient Attention (BEA) and Frequency-Enhanced Feed-Forward Network (FEFFN) for enhanced feature representation.

Traditional AIFI (Attention-based Intra-Feature Interaction) encoders face numerous limitations when processing special vehicle image features, mainly manifested as unbalanced attention allocation for targets under complex backgrounds, limited long-range dependency modeling capabilities, and insufficient frequency-domain information processing, leading to inadequate accuracy and robustness when recognizing special vehicles and their key components (such as observation chambers, communication equipment, etc.). Addressing these challenges, we propose the Dual-Domain Feature Encoder (DDFE), with its structure shown in Figure 7. This encoder combines Bipolar Efficient Attention (BEA) and Frequency-Enhanced Feed-Forward Network (FEFFN), significantly enhancing the system’s recognition capabilities for special vehicles under various environmental conditions.

The DDFE adopts a dual-stage design approach, achieving comprehensive extraction and refined modulation of feature information. Its overall workflow can be summarized through the following mathematical expression:

(11) $$X_{out}=N_{2}(X+D_{2}{(F}_{FN}{(N}_{1}(X+D_{1}{(F}_{PLA}(X))))))$$

where X represents input features, $F_{PLA}$ represents bipolar linear attention operations, $F_{FN}$ represents frequency-domain modulation feed-forward networks, $D_{1}$ and $D_{2}$ represent dropout operations, and $N_{1}$ and $N_{2}$ represent layer normalization. The encoder first achieved adaptive weight allocation for features through BEA, focusing on feature representation and structural relationships of large armored vehicles; then, it modulated and enhanced features in the frequency domain through FEFFN, capturing more detail information; finally, it maintained information flow stability through residual connections and normalization.

BEA achieves efficient computation and precise feature extraction through innovative bipolar representation and linear attention mechanisms. Its attention computation process can be expressed through the following key formulas:

(12) $$\tilde{Q}=\left[R{\left(Q/s\right)}^{p},R{\left(-Q/s\right)}^{p}\right]$$

(13) $$\tilde{K}=\left[R{\left(K/s\right)}^{p},R{\left(-K/s\right)}^{p}\right]$$

where Q and K represent query and key matrices, respectively, s is a learnable scale parameter, p is a learnable polarity power parameter, R represents ReLU activation function, and $\left[\cdot ,\cdot \right]$ represents feature concatenation operation. Through this positive–negative polarity decomposition, the network can simultaneously capture complementary representations of features, enhancing expression capabilities for vehicle details. BEA’s computation adopts an efficient linear form:

(14) $$Z_{sim}=\frac{{\hat{Q}}_{sim}\cdot \left({\hat{K}}^{T}\cdot V_{1}/\sqrt{n}\right)}{{\hat{Q}}_{sim}\cdot {\bar{K}}^{T}+\epsilon }$$

(15) $$Z_{opp}=\frac{{\hat{Q}}_{opp}\cdot \left({\hat{K}}^{T}\cdot V_{2}/\sqrt{n}\right)}{{\hat{Q}}_{opp}\cdot {\bar{K}}^{T}+\epsilon }$$

where ${\tilde{Q}}_{sim}$ and ${\tilde{Q}}_{opp}$ represent similar and opposite polarity query representations, respectively, $V_{1}$ and $V_{2}$ are two sub-parts of the value matrix, K represents the average representation of keys, n is the number of keys, and ϵ is a stability factor. The final attention output was modulated through gating mechanisms and position enhancement:

(16) $$X_{out}=G⊙\left[Z_{sim},Z_{opp}\right]+F_{DW}\left(V\right)$$

where G is the gating factor, ⊙ represents element-wise multiplication, and $F_{DW}$ represents depth-wise separable convolution operations.

Frequency-Enhanced Feed-Forward Network (FEFFN) achieves effective extraction of special vehicle detail features through selective enhancement of features in the frequency domain space. The overall mathematical expression of FEFFN is as follows:

(17) $$Y=F_{M}{(W}_{2}(\sigma {(W}_{1}(X))))$$

where $W_{1}$ and $W_{2}$ represent weights of two convolutional layers, respectively, σ represents nonlinear activation function, and $F_{M}$ represents frequency-domain modulation operations. The core mathematical expression of the frequency-domain modulation module Window Frequency Modulation is

(18) $$F_{M}\left(X\right)=IFFT(FFT(W(X))⊙\Omega )$$

where W represents window rearrangement operations, reorganizing features into local windows; FFT and IFFT represent two-dimensional fast Fourier transform and its inverse transform, respectively; Ω is a learnable complex weight parameter; and ⊙ represents complex multiplication. Through selective modulation of features in the frequency domain, FEFFN can enhance texture, edge, and structural detail features of special vehicles while suppressing background noise and interference, making the network more robust to environmental variations.

The DDFE, through innovative design of bipolar linear attention and frequency-domain modulation, demonstrates significant advantages compared to traditional AIFI encoders. The bipolar linear attention mechanism achieves more precise feature weight allocation, significantly improving positioning accuracy for vehicle key components; frequency-domain modulation technology enhances the model’s capability to capture feature details, enabling the system to better recognize vehicle observation chambers, communication equipment, and other key components; the high-efficiency computational characteristics of linear attention significantly reduce computational complexity, enabling the model to maintain high accuracy while possessing stronger real-time performance; finally, the overall design of the encoder improves adaptability to complex environmental factors, enabling the system to maintain stable performance under various lighting conditions, partial occlusion, and complex backgrounds.

### 3.4. Spatial-Channel Fusion Upsampling Block (SCFUB)

**Figure 8:**
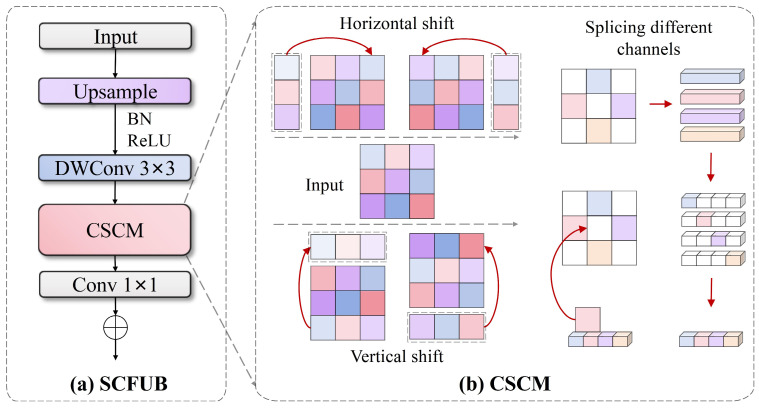
Spatial-Channel Fusion Upsampling Block (SCFUB) employing depth-wise separable convolution and Cross-Spatial Channel Mixer (CSCM) for high-fidelity feature upsampling.

Traditional convolutional downsampling and nearest neighbor interpolation upsampling face obvious limitations when processing multi-scale features of special vehicles, mainly manifested as the following: severe feature accuracy loss during upsampling, limited detail recovery capabilities, insufficient information interaction between different channels, and difficulty maintaining feature spatial consistency. These deficiencies are particularly prominent when recognizing observation chambers, sensor devices, and other key components of large armored vehicles, leading to blurred boundaries, missing small targets, and inaccurate fine structure recognition. Addressing these challenges, we propose the Spatial-Channel Fusion Upsampling Block (SCFUB), with its structure shown in Figure 8. This module significantly enhances feature fidelity and spatial consistency during upsampling while maintaining computational efficiency through combining depth-wise separable convolution with channel shift mixing techniques, effectively addressing fine recognition issues of special vehicles in complex environments.

The SCFUB upsampling module achieves efficient and refined feature upsampling through a three-stage workflow of “scale expansion—channel reorganization—feature mixing”. Its overall mathematical expression can be formalized as follows:

(19) $$Y=P\left(S\left(U\left(X⊙W_{d}\right),g\right)\right)$$

where X and Y represent input and output feature maps, respectively, U represents scale expansion operations, ⊙ represents depth convolution operations, $W_{d}$ represents depth convolution kernels, S represents channel reorganization function, P represents Cross-Spatial Channel Mixer (CSCM), C represents pointwise convolution, and g represents the number of groups. The workflow first achieved feature map scale expansion through nearest neighbor interpolation and then used depth-wise separable convolution for feature enhancement, during which channel reorganization and shift mixing operations were employed to enhance cross-channel interaction.

CSCM achieves feature enhancement and mixing through efficient channel segmentation and spatial shift operations. Its mathematical expression can be succinctly represented as follows:

(20) $$S\left(X\right)=\left[R_{s}^{h+}\left(X_{1}\right),R_{s}^{h-}\left(X_{2}\right),R_{s}^{w+}\left(X_{3}\right),R_{s}^{w-}\left(X_{4}\right)\right]$$

where $X=\left[X_{1},X_{2},X_{3},X_{4}\right]$ represents features equally divided along the channel dimension, $R_{s}^{h+}$, $R_{s}^{h-}$, $R_{s}^{w+}$, and $R_{s}^{w-}$ represent positive and negative direction circular shift operations in height and width dimensions, respectively, s represents shift size, and $\left[\cdot \right]$ represents channel concatenation. This operation first divided the feature map into four sub-blocks along the channel dimension and then applied circular shifts in different directions to different spatial dimensions for these sub-blocks, finally recombining them to form mixed features. This design enables different channels to perceive different spatial position information, effectively enhancing feature expression capabilities and spatial perception while maintaining extremely low computational overhead.

## 4. Experiments

### 4.1. Datasets

The VisDrone2019 [34] dataset is a large-scale unmanned aerial vehicle (UAV) aerial target detection benchmark dataset constructed by the AISKYEYE team at Tianjin University. This dataset contains 10,209 static images and 288 video clips (totaling 261,908 frames), covering diverse scenarios from 14 different cities in China, including urban and rural environments. The dataset provides over 2.6 million precisely annotated bounding boxes with target categories including pedestrians, vehicles, bicycles, tricycles, and 10 other classes. This dataset was collected under different UAV platforms, scene conditions, weather, and lighting conditions, providing rich training and testing samples for aerial small target detection research.

The BDD100K [35] dataset is currently the largest-scale autonomous driving video dataset released by the Berkeley DeepDrive team. This dataset contains 100,000 high-resolution video clips, each approximately 40 s long (720p, 30 fps), representing over 1000 h of driving experience and over 100 million frames. The dataset features geographical, environmental, and weather diversity, covering different road scenarios across multiple regions in the United States, including urban streets, residential areas, and highways.

### 4.2. Implementation Details and Training Configuration

Experiments were conducted on a server configured with Windows 10 operating system, using Python 3.10.16 and PyTorch 2.3.0 deep learning framework. Hardware configuration includes RTX 3090 GPU and CUDA 11.8. The AdamW optimizer was used during training with a learning rate set to 0.0001 and a batch size of 8. Model training was conducted for a total of 300 epochs. The weight decay coefficient was set to 0.0001. All experiments used the same random seed to ensure result reproducibility. Other parameters adopted RT-DETR default settings. FPS evaluation was conducted on an RTX 3090 GPU using an input resolution of 640 × 640 pixels with batch size 1. All inference timing measurements exclude data loading and post-processing time to ensure fair comparison across different methods.

### 4.3. Evaluation Metrics

This paper employs standard evaluation metrics from the object detection domain to comprehensively assess the performance of the HSF-DETR framework. Main evaluation metrics include the following: Precision (P) for measuring detection result accuracy, i.e., the proportion of correct detections among all detection boxes; Recall (R) for evaluating the model’s target detection capability, i.e., the proportion of correctly detected targets among all real targets; Average Precision mAP50 represents average precision at the IoU threshold of 0.5, which is the most commonly used performance metric in object detection; mAP50-95 represents average precision across IoU thresholds from 0.5 to 0.95, providing stricter evaluation of detection box localization accuracy. Additionally, experiments also recorded model computational complexity metrics, including floating-point operations (GFLOPs) and parameter count (Params), to evaluate model computational efficiency and practicality. The comprehensive evaluation of these metrics can fully reflect HSF-DETR’s performance in special vehicle detection tasks.

### 4.4. Ablation Studies and Component Analysis

#### 4.4.1. HyperSFM Module Component Analysis

To validate the effectiveness of our proposed HyperSFM module, we conducted detailed ablation experiments. The experiments systematically analyzed the contributions of each key component in HyperSFM, including the independent roles of SFM and HRA and their collaborative effects. Ablation experiments were based on the RT-DETR baseline architecture, quantifying the improvement effect of each component on overall performance by gradually adding different modules.

As shown in Table 1, the HyperSFM module significantly improves detection performance while maintaining reasonable computational overhead. Specifically, compared to the baseline RT-DETR model, adding the SFM module alone increased mAP50 by 0.6% and mAP50-95 by 1.1%; the introduction of HRA further enhanced the model’s capability for complex spatial relationship modeling, achieving mAP50-95 of 67.3%. When SFM and HRA work collaboratively to form the complete HyperSFM, the model achieves optimal performance, improving by 1.0% and 1.8%, respectively, compared to the baseline model. We evaluated the impact of threshold parameter τ on detection performance across values from 2 to 8. Results show that τ = 4 achieves optimal performance with mAP50 of 94.5%. Lower values (τ = 2) result in overly restrictive hyperedge formation, limiting the capture of broader spatial relationships and reducing mAP50 to 92.8%. Higher values (τ = 6, 8) create excessive connections, introducing noise and reducing precision, with mAP50 dropping to 93.9% and 93.4%. The optimal τ = 4 balances local spatial coherence with computational efficiency, enabling effective capture of vehicle component relationships while avoiding over-connection artifacts. Although HyperSFM introduces moderate computational overhead and parameter increase, the significant performance improvements it brings fully validate the effectiveness of the hypergraph-enhanced feature modulation mechanism in special vehicle detection tasks.

#### 4.4.2. Overall Framework Ablation Study

To validate the effectiveness of our proposed HSF-DETR framework, we conducted comprehensive ablation experiments on special vehicle detection tasks. The experiments systematically analyzed the independent contributions and collaborative effects of the four core innovative modules in HSF-DETR, including Cascaded Spatial Feature Network (CSFNet), Hypergraph-enhanced Spatial Feature Modulation (HyperSFM), Dual-Domain Feature Encoder (DDFE), and Spatial-Channel Fusion Upsampling Block (SCFUB). Ablation experiments were based on the RT-DETR-R18 baseline architecture, quantifying the improvement effect of each component on overall detection performance by gradually adding different module combinations, with experimental results shown in Table 2.

As shown in the table, each innovative module of HSF-DETR produces significant performance improvements, validating the effectiveness of each module design. Specifically, the CSFNet module enhances feature extraction capabilities, improving mAP50 and mAP50-95 by 1.1% and 2.4%, respectively, while significantly reducing computational overhead, validating the high efficiency of the Cascaded Spatial Feature Network; the HyperSFM module achieves mAP50-95 of 67.8% through hypergraph-enhanced feature modulation mechanisms, demonstrating the importance of high-order feature correlation modeling; the DDFE module and SCFUB module improve mAP50 to 94.8% and 94.5%, respectively, validating the effectiveness of dual-domain feature encoding and Spatial-Channel Fusion Upsampling. When all innovative modules work collaboratively, the complete HSF-DETR framework achieves optimal performance, with mAP50 and mAP50-95 improving by 3.1% and 4.6%, respectively, compared to the baseline RT-DETR-R18 model, while maintaining reasonable computational complexity, fully demonstrating HSF-DETR’s excellent performance and practical value in special vehicle detection tasks.

### 4.5. Comparative Experiments

#### 4.5.1. Backbone Network Comparison

To validate the effectiveness of our proposed CSFNet backbone network, we conducted comprehensive comparison experiments with multiple mainstream backbone networks on special vehicle detection tasks. The experiments selected representative backbone network architectures, including classic ResNet, lightweight Fasternet and EfficientViT, VanillaNet, Swin Transformer, and other different types of feature extraction networks. All comparison experiments were based on the RT-DETR detection framework, fairly evaluating the feature extraction capabilities and detection performance of each network by replacing different backbone networks, with experimental results shown in Table 3.

As shown in Table 3, our proposed CSFNet backbone network demonstrates significant advantages in accuracy and efficiency balance. Specifically, compared to the baseline rtdetr-ResNet, the CSFNet achieved higher detection accuracy with mAP50 and mAP50-95 reaching 94.6% and 68.4%, respectively, while significantly reducing computational complexity to 47.9 GFLOPs and parameter count from 19.87 M to 14.49 M. Compared to lightweight networks Fasternet and EfficientViT, the CSFNet achieved significant accuracy improvements with slightly higher computational overhead. More importantly, compared to large networks VanillaNet and SwinTransformer, the CSFNet not only improved mAP50 by 0.6% and 0.8%, respectively, but also achieved 2–3 times performance optimization in computational efficiency, fully validating CSFNet’s efficient feature extraction capabilities through Cross-Efficient Convolutional Gating modules and superior adaptability to special vehicle detection tasks.

#### 4.5.2. Feature Fusion Network Comparison

To validate the effectiveness of our proposed HyperSFM feature fusion module, we conducted comprehensive comparison experiments with multiple advanced feature fusion networks on special vehicle detection tasks. The experiments selected representative feature fusion methods, including traditional CCFF, lightweight Slimmeck, efficient HSPFN, bidirectional feature fusion BiFPNs, multi-scale adaptive MAFPNs, and other different types of feature fusion architectures. All comparison experiments were based on the RT-DETR detection framework, fairly evaluating the multi-scale feature integration capabilities and detection performance of each method by replacing different feature fusion modules, with experimental results shown in Table 4.

As shown in Table 4, our proposed HyperSFM module demonstrates significant performance advantages in feature fusion effectiveness. Specifically, compared to the baseline CCFF method, HyperSFM achieved significant improvements with mAP50 and mAP50-95 reaching 94.5% and 67.8%, respectively, improving by 1.0% and 1.8%. Compared to lightweight fusion methods Slimmeck and HSPFN, HyperSFM achieved improvements of 1.7% and 1.6% in mAP50, respectively, and even greater improvements of 3.0% and 2.9% in mAP50-95. Computational cost analysis reveals that our HyperSFM module introduces moderate overhead compared to traditional fusion methods. Specifically, HyperSFM requires 61.2 GFLOPs compared to FPN’s 56.9 GFLOPs and BiFPN’s 64.3 GFLOPs. The additional 4.3 GFLOPs are primarily attributed to hypergraph convolution operations and cross-position aggregation. However, this computational investment yields significant accuracy improvements: +1.0% mAP50 over FPN and +0.5% over BiFPN. The computational complexity scales linearly with the number of feature nodes, making it suitable for various input resolutions. More importantly, compared to advanced BiFPN and MAFPN methods, HyperSFM not only improved accuracy by 0.5% and 1.3%, respectively, but also maintained reasonable computational overhead, fully validating the excellent capability of the hypergraph-enhanced feature modulation mechanism in modeling high-order feature correlations and achieving adaptive multi-scale fusion, providing strong technical support for the precise detection of special vehicle key components.

#### 4.5.3. Comparing Different Mainstream SOTA Models

Current object detection methodologies exhibit significant contradictions and ongoing debates regarding optimal architectural choices. Traditional CNN-based approaches like YOLO series prioritize speed and efficiency, achieving real-time performance through single-stage detection paradigms. However, these methods suffer from inherent limitations including dependency on non-maximum suppression (NMS) post-processing, which introduces computational overhead and potential accuracy degradation. Recent studies demonstrate that NMS can reduce detection performance by 2–5% while adding 10–20 ms latency per inference. In contrast, Transformer-based detectors like DETR eliminate NMS requirements through end-to-end training but face criticism for high computational costs and slow convergence. RT-DETR attempts to bridge this gap, yet debates persist regarding whether CNN or Transformer architectures provide superior feature extraction for complex scenes. Furthermore, the hypergraph computing community presents conflicting approaches: semantic-based methods (Hyper-YOLO) emphasize feature similarity in embedding space, while our geometric-driven approach prioritizes spatial relationships. This fundamental difference reflects an ongoing debate about whether high-order relationships should be modeled through learned semantic representations or explicit geometric constraints. These contradictions highlight critical knowledge gaps in balancing accuracy, efficiency, and adaptability across diverse detection scenarios, which our HSF-DETR framework specifically addresses through its unified architecture.

To validate the effectiveness of our proposed HSF-DETR framework, we conducted comprehensive comparison experiments with multiple mainstream object detection methods on special vehicle detection tasks. The experiments covered three representative detection architectures: single-stage detection YOLO series methods (YOLOv5m, YOLOv8m, YOLOv9m, YOLOv10m, YOLOv11m); two-stage detection Faster R-CNN; and advanced DETR-based detectors (DINO, DEIM-D-Fine-M, D-Fine-M, RT-DETR-L, RT-DETR-r50, RT-DETR-r34, RT-DETR-r18) and other different types of object detection methods. All comparison experiments were conducted under the same dataset and evaluation protocols to ensure the fairness and comparability of experimental results, with results shown in Table 5.

RT-DETR was selected as our primary baseline for several compelling reasons specific to this research context. First, RT-DETR represents the state-of-the-art in real-time DETR architectures, achieving optimal balance between accuracy and speed essential for practical special vehicle detection applications. Unlike DINO Deformable-DETR, which suffers from slower inference speeds (20+ FPS), RT-DETR maintains real-time performance (70+ FPS) while preserving detection accuracy. Second, RT-DETR’s hybrid encoder design provides an ideal foundation for integrating our proposed modules, allowing a fair comparison of individual component contributions. Third, compared to YOLO variants (YOLOv9, YOLOv11), which require extensive post-processing like NMS, RT-DETR’s end-to-end architecture aligns better with our design philosophy. Nevertheless, we have included comprehensive comparisons with YOLOv9, YOLOv10, YOLOv11, and DINO Deformable-DETR in Table 5 to demonstrate the broad applicability and superiority of our approach across different architectural paradigms.

As shown in Table 5, our proposed HSF-DETR achieved significant performance improvements in detection accuracy, fully validating the effectiveness of the framework design. Specifically, compared to the best-performing RT-DETR-r18 baseline model among similar DETR architectures, HSF-DETR achieved improvements of 3.1% and 4.6% in mAP50 and mAP50-95, respectively. Compared to the best-performing YOLOv8m in the YOLO series, HSF-DETR achieved substantial improvements of 4.8% and 5.4% in mAP50 and mAP50-95. More importantly, compared to advanced DETR methods such as DINO and RT-DETR-r50, HSF-DETR not only improved accuracy by 4.1% and 4.2% as well as 2.4% and 2.8%, respectively, but also demonstrated excellent computational efficiency, requiring only 59.7 GFLOPs and 18.07 M parameters, significantly lower than RT-DETR-r50’s parameters, fully demonstrating that HSF-DETR achieves optimal balance between accuracy and efficiency in special vehicle detection tasks through innovative designs such as Cascaded Spatial Feature Networks, hypergraph-enhanced feature modulation, dual-domain feature encoding, and Spatial-Channel Fusion Upsampling. In terms of inference speed, HSV-DETR achieved 70.2 FPS on RTX 3090. Our method achieved competitive real-time performance and was comparable to other mainstream methods, confirming its practical applicability to real-time special vehicle inspection scenarios.

**Figure 9:**
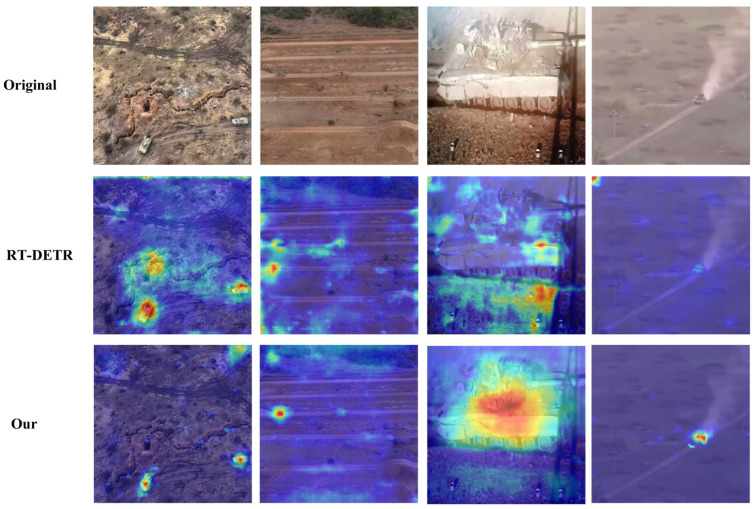
Attention heatmap visualization comparison between RT-DETR and HSF-DETR.

To validate the effectiveness of our proposed HSF-DETR framework in complex environment special vehicle detection tasks and the advantages of attention mechanisms, we conducted detailed attention heatmap visualization comparison experiments. The experiments selected representative complex special vehicle detection scenarios, including armored vehicles under complex terrain environments, armored equipment in open areas, armored vehicles in field environments, and long-range targets under low-visibility conditions and other typical challenging application scenarios. Through comparing attention heatmaps with the baseline model RT-DETR, we deeply explored the improvement effects of each innovative module of HSF-DETR on model attention distribution and target-focusing capabilities, with visualization results shown in Figure 9.

As shown in Figure 9, HSF-DETR demonstrates significant advantages in attention distribution precision and target-focusing capabilities compared to RT-DETR. Specifically, in complex terrain scenarios (first column), RT-DETR’s attention distribution is relatively scattered with obvious background interference responses, while HSF-DETR achieves more concentrated and precise attention focusing through the HyperSFM module’s hypergraph-enhanced feature modulation mechanism, effectively suppressing complex background interference. In open-area detection scenarios (second column), HSF-DETR demonstrates stronger target positioning capabilities, with attention heatmaps showing clearer target contours and higher activation intensity. In field environment armored vehicle detection tasks (third column), HSF-DETR produces more concentrated and stable attention responses through the dual-domain feature encoding of the DDFE module and cascaded spatial feature extraction of CSFNet, successfully overcoming challenges of lighting variations and shadow interference. In low-visibility long-range target scenarios (fourth column), HSF-DETR, leveraging the high-quality feature upsampling capabilities of the SCFUB module, can still produce clear attention activation even under weak target information conditions, fully validating HSF-DETR’s stronger feature expression capabilities and target perception accuracy under complex environments.

### 4.6. Cross-Domain Generalization Analysis

To validate the generalization capabilities and robustness of our proposed HSF-DETR framework, we conducted cross-domain generalization experiments on two other representative object detection datasets. The experiments selected the VisDrone2019 UAV aerial dataset and the BDD100K autonomous driving dataset as evaluation benchmarks. These two datasets represent UAV aerial small target dense detection tasks and multi-category target detection tasks in complex traffic scenarios, respectively, which differ significantly from our special vehicle detection tasks in perspective, target scale, and scene complexity. Through comparison experiments with the baseline model RT-DETR-R18, we comprehensively evaluated the adaptability and effectiveness of each innovative module of HSF-DETR in different application domains, with experimental results shown in Table 6.

As shown in Table 6, HSF-DETR achieved consistent performance improvements on both cross-domain datasets, fully validating the good generalization capabilities of the framework design. Specifically, on the VisDrone2019 dataset, compared to the baseline RT-DETR-R18 (mAP50: 47.4%, mAP50-95: 29.2%), HSF-DETR achieved mAP50 improvement from 47.4% to 48.3%, Precision from 61% to 62.9%, and Recall from 46.6% to 47.1%, demonstrating the effectiveness of CSFNet and HyperSFM modules in handling aerial small target detection tasks. On the BDD100K dataset, HSF-DETR also performed excellently, with mAP50 improving from 49.1% to 50.3%, mAP50-95 from 31.9% to 32.8%, and Precision and Recall improving by 0.8% and 0.7%, respectively, proving the robustness of DDFE and SCFUB modules under complex traffic scenarios. It is worth noting that, despite significant differences between these two datasets and special vehicle detection tasks in target types and scene characteristics, HSF-DETR still maintained stable performance improvements, fully demonstrating that the proposed cascaded spatial feature networks, hypergraph-enhanced feature modulation, and other innovative designs possess good cross-domain adaptability and broad application potential.

### 4.7. Visualization Results

#### 4.7.1. Special Vehicle Detection Visualization

**Figure 10:**
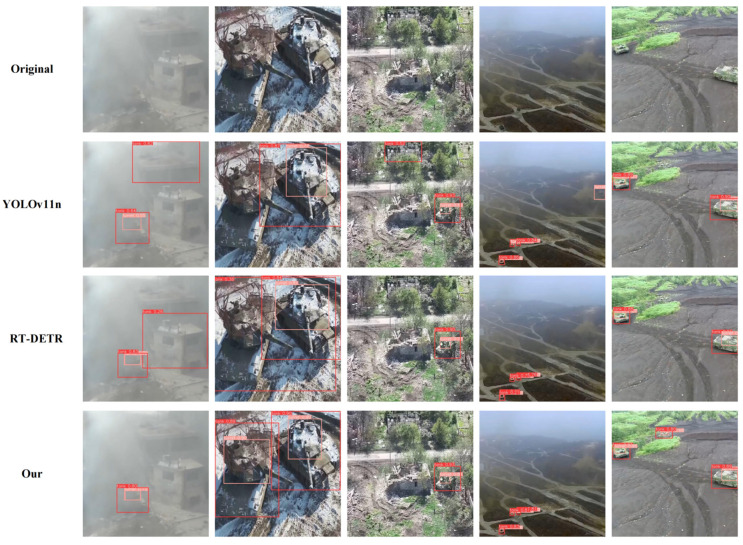
Qualitative detection results comparison on special vehicle dataset.

To validate the effectiveness of our proposed HSF-DETR framework in complex environment special vehicle detection, we conducted detailed visualization comparison experiments. The experiments selected challenging special vehicle detection scenarios, including low-visibility smoke environments, armored vehicles in complex terrain, military equipment under vegetation occlusion, long-range aerial perspectives, and multi-target dense distribution, and other typical application scenarios. Through intuitive detection effect comparisons with current mainstream detection methods YOLOv11n and RT-DETR, we comprehensively evaluated HSF-DETR’s detection performance under different environmental conditions and target states, with visualization results shown in Figure 10.

As shown in the figure, HSF-DETR demonstrates excellent detection performance across various complex scenarios, significantly outperforming comparison methods. Specifically, in low-visibility scenarios (first column), both YOLOv11n and RT-DETR exhibit significant false detection cases, while HSF-DETR achieves precise target positioning and recognition through the dual-domain feature enhancement capabilities of the DDFE module. In complex terrain scenarios (second and third columns), facing partial occlusion and the complex background interference of armored vehicles, YOLOv11n and RT-DETR both show varying degrees of false detections and missed detections, while HSF-DETR, leveraging the hypergraph-enhanced feature modulation mechanism of the HyperSFM module, can accurately capture high-order spatial relationships between various vehicle key components, achieving more precise and complete target detection. In long-range detection scenarios (fourth and fifth columns), HSF-DETR, through the cascaded spatial feature extraction of CSFNet and high-quality feature upsampling of SCFUB, effectively addresses small target detection challenges, demonstrating higher detection accuracy and lower false detection rates compared to other methods, fully validating HSF-DETR’s technical advantages and practical value in special vehicle detection tasks.

**Figure 11:**
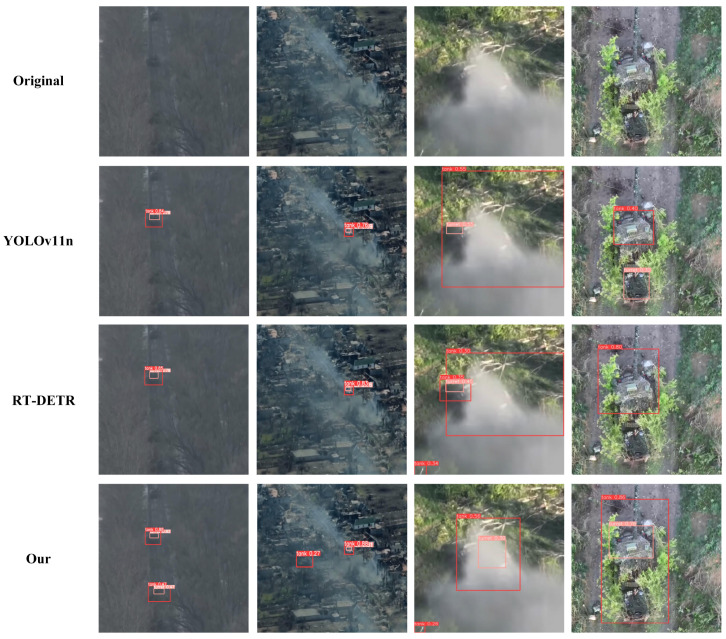
Visualization of detection failure cases.

Through the analysis in Figure 10, it can be observed that our model demonstrates outstanding detection performance in complex environments regarding partial occlusion, multi-scale variations, and light camouflage. However, the model exhibits certain limitations in scenarios with low illumination, heavy occlusion, and high camouflage. Figure 11 presents the visualization of detection failure cases. As shown in Figure 11, in the first column of low-light aerial scenarios containing five tanks, YOLOv11n, RT-DETR, and HSF-DETR all missed detections, while HSF-DETR successfully detected two tanks, demonstrating superior detection capability in low-light conditions. In the second column of low-light scenarios with smoke interference, YOLOv11n and RT-DETR only detected one tank, whereas HSF-DETR identified all tanks but failed to detect the turret of the second tank, indicating its better adaptability in complex smoke-affected low-light environments. In the third column of severely smoke-obscured scenarios, YOLOv11n and RT-DETR showed both missed and false detections, while HSF-DETR correctly detected tanks and turrets though with some false positives, exhibiting superior target detection and localization capabilities. In the fourth column of high-camouflage environments, YOLOv11n produced missed and false detections, RT-DETR showed missed detections with poor localization, and HSF-DETR successfully identified tanks and turrets, though requiring further improvement in localization accuracy, demonstrating enhanced feature extraction and target recognition capabilities in high-camouflage scenarios.

#### 4.7.2. BDD100K Dataset Visualization Analysis

**Figure 12:**
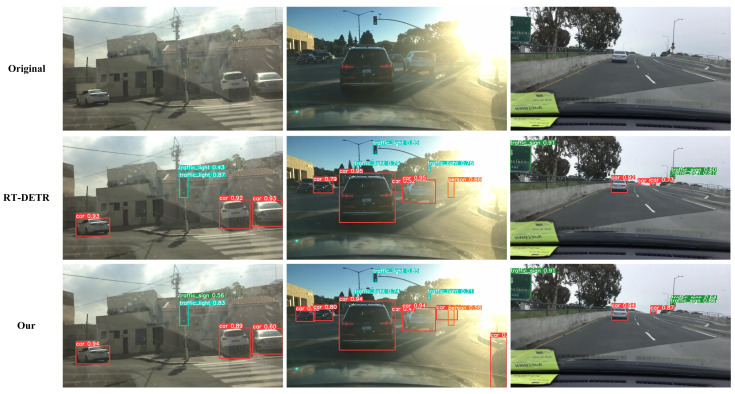
Visualization comparison between RT-DETR and HSF-DETR on the BDD100K dataset.

To validate the generalization capabilities and detection performance of our proposed HSF-DETR framework in complex traffic scenarios, we conducted detailed visualization comparison experiments on the BDD100K autonomous driving dataset. The experiments selected representative urban traffic scenarios, including complex intersection environments, strong lighting interference conditions, highway multi-lane scenarios, and other typical autonomous driving application scenarios. Through intuitive detection effect comparisons with the baseline model RT-DETR, we comprehensively evaluated HSF-DETR’s accuracy performance and robustness when processing multi-category target detection tasks, particularly the recognition accuracy for key traffic elements such as vehicles, traffic signs, and pedestrians, with visualization results shown in Figure 12.

As shown in the figure, HSF-DETR demonstrates superior detection performance compared to baseline methods across various complex traffic scenarios. Specifically, in complex intersection scenarios (first column), HSF-DETR not only accurately detected all vehicle targets with generally higher detection confidence (car target confidence reaching 0.94, 0.89, 0.60) but also achieved more precise and stable detection of traffic signs (traffic-light and traffic-sign confidence reaching 0.83 and 0.56, respectively). In strong lighting interference environments (second column), facing challenges such as backlighting and glare, HSF-DETR achieved more accurate detection and positioning of front vehicles through the dual-domain feature enhancement capabilities of the DDFE module, while RT-DETR exhibits missed detections under the same conditions. In highway multi-lane scenarios (third column), HSF-DETR, leveraging the hypergraph-enhanced feature modulation mechanism of the HyperSFM module and efficient feature extraction capabilities of CSFNet, better handles long-range small targets and densely distributed vehicles, with detection confidence and target coverage rates both superior to baseline methods, fully validating HSF-DETR’s technical advantages and good generalization performance in cross-domain applications.

#### 4.7.3. VisDrone2019 Dataset Visualization Analysis

**Figure 13:**
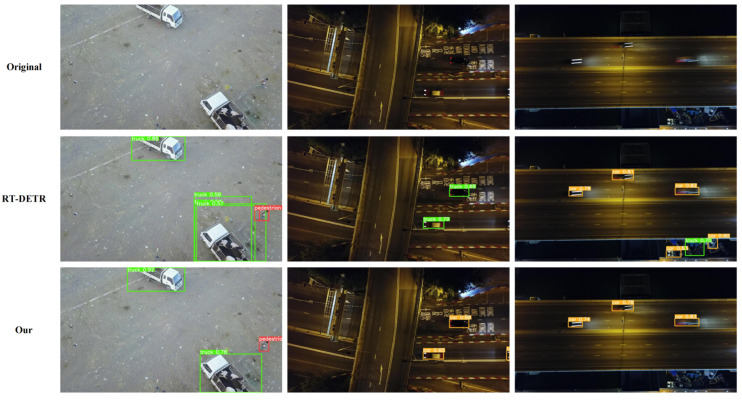
Visualization comparison between RT-DETR and HSF-DETR.

To validate the effectiveness and robustness of our proposed HSF-DETR framework in aerial small target detection tasks, we conducted detailed visualization comparison experiments on the VisDrone2019 UAV aerial dataset. The experiments selected representative UAV aerial scenarios, including urban road vehicle detection from high-altitude bird’s-eye view, multi-target distribution scenarios in complex outdoor traffic environments, and challenging detection tasks under nighttime low-light conditions and other typical aerial application scenarios. Through intuitive detection effect comparisons with the baseline model RT-DETR, we comprehensively evaluated HSF-DETR’s detection performance under small targets, dense targets, and adverse lighting conditions, with visualization results shown in Figure 13.

As shown in the figure, HSF-DETR demonstrates significant advantages over baseline methods across various aerial scenarios. Specifically, in high-altitude bird’s-eye view vehicle detection scenarios (first column), HSF-DETR not only accurately detected all truck targets (truck confidence reaching 0.92 and 0.78, respectively) but also successfully recognized pedestrian targets, while RT-DETR showed obvious false detection phenomena in the same scenarios, particularly insufficient detection capabilities for long-range small targets. In complex outdoor traffic environments (second column), facing distributed vehicle targets and complex light–shadow variations, HSF-DETR achieved more precise and complete target detection through the hypergraph-enhanced feature modulation of the HyperSFM module and efficient feature extraction capabilities of CSFNet, with more accurate detection box positioning. In nighttime low-light scenarios (third column), HSF-DETR effectively overcame challenges of insufficient lighting and noise interference through the dual-domain feature enhancement mechanism of the DDFE module, demonstrating higher detection accuracy and lower false detection rates compared to RT-DETR, fully validating HSF-DETR’s technical advantages in aerial small target detection tasks and strong adaptability to complex environmental conditions.

## 5. Conclusions

This paper addresses key challenges in special vehicle detection under complex environments, including insufficient feature extraction, inadequate multi-scale fusion, unbalanced attention allocation, and upsampling information loss, and it proposes the HSF-DETR detection framework. Through the collaborative design of four innovative modules, the framework achieves the high-precision detection of special vehicles and their key components: CSFNet backbone network enhances long-range dependency modeling capabilities through CECG modules; HyperSFM feature fusion network utilizes hypergraph structures to achieve high-order feature correlation modeling; DDFE improves feature expression capabilities through bipolar attention and frequency-domain modulation; SCFUB upsampling module effectively maintains feature fidelity.

Experimental results demonstrate that HSF-DETR achieved significant performance improvements in special vehicle detection tasks, with mAP50 and mAP50-95 reaching 96.6% and 70.6%, respectively, improving by 3.1% and 4.6% compared to baseline methods. In terms of computational efficiency, this method requires only 59.7 GFLOPs and 18.07 M parameters, achieving a good balance between accuracy and efficiency. Generalization experiments demonstrate the method’s adaptability and robustness in different application scenarios.

Future work will focus on the following directions: (1) further optimizing model structure to improve real-time performance; (2) expanding dataset scale and scene diversity; (3) exploring multi-modal fusion technologies to enhance detection capabilities under complex environments; and (4) applying the method to broader military and civilian special vehicle detection scenarios.

## Figures and Tables

**Table 1:** Ablation study results for HyperSFM module components.

RT-DETR	SFM	HRA	GFLOPs	Params (M)	mAP50 (%)	mAP50-95 (%)
√			56.9	19.87	93.5	66.0
√	√		58.1	19.98	94.1	67.1
√		√	60.4	21.72	93.9	67.3
√	√	√ (τ = 4)	61.2	21.92	94.5	67.8
√	√	√ (τ = 6)	61.2	21.92	93.9	66.9
√	√	√ (τ = 8)	61.2	21.92	93.4	66.1
√	√	√ (τ = 2)	61.2	21.92	92.8	65.2

**Table 2:** Comprehensive ablation study of HSF-DETR framework components.

Model	CSFNet	HyperSFM	DDFE	SCFUB	GFLOPs	Params (M)	P (%)	R (%)	mAP50 (%)	mAP50-95 (%)
1.base					56.9	19.87	95.8	90.9	93.5	66.0
2	√				47.9	14.48	96.0	92.3	94.6	68.4
3		√			61.2	21.92	96.2	92.9	94.5	67.8
4			√		57.2	20.04	95.9	92.5	94.8	67.1
5				√	58.0	20.01	95.7	92.3	94.5	67.4
6	√	√			55.1	16.76	96.6	93.7	95.5	68.2
7	√		√		48.2	14.66	96.3	93.4	95.4	68.3
8		√		√	65.5	23.05	96.1	94.2	95.4	68.5
9	√	√	√		55.4	16.94	96.7	94.5	96.2	69.0
10		√	√	√	65.7	23.22	97.2	93.9	96.0	68.9
11.ours	√	√	√	√	59.7	18.07	97.4	95.1	96.6	70.6

**Table 3:** Performance comparison of different backbone networks on special vehicle detection.

Model	GFLOPs	Params (M)	P (%)	R (%)	mAP50 (%)	mAP50-95 (%)
rtdetr-Resnet	56.9	19.87	95.8	90.9	93.5	66.0
rtdetr-Fasternet [36]	28.5	10.81	95.2	89.8	92.8	64.5
rtdetr-EfficientViT [37]	27.6	10.80	95.1	89.5	92.5	64.2
rtdetr-VanillaNet [38]	110.1	21.71	96.2	91.5	94.0	67.5
rtdetr-SwinTransformer [39]	98.4	36.61	96.1	91.2	93.8	67.2
rtdetr-CSFNet	47.9	14.48	96.0	92.3	94.6	68.4

**Table 4:** Comparative evaluation of different feature fusion networks.

Model	GFLOPs	Params (M)	P (%)	R (%)	mAP50 (%)	mAP50-95 (%)
rtdetr-CCFF	56.9	19.87	95.8	90.9	93.5	66.0
rtdetr-Slimmeck [40]	53.2	19.3	95.2	89.5	92.8	64.8
rtdetr-HSPFN [41]	53.3	18.11	95.3	89.6	92.9	64.9
rtdetr-BiFPN [42]	64.3	20.3	96.0	91.5	94.0	67.0
rtdetr-MAFPN [43]	56.3	22.92	95.5	90.2	93.2	65.5
rtdetr-HyperSFM	61.2	21.92	96.2	92.9	94.5	67.8

**Table 5:** Performance comparison with state-of-the-art detection methods.

Model	Type	GFLOPs	Params (M)	P (%)	R (%)	mAP50 (%)	mAP50-95 (%)	FPS
YOLOv5m [44]	One-Stage	64.0	25.0	94.2	88.6	90.8	63.8	125.7
YOLOv8m [45]	One-Stage	78.7	25.8	95.1	89.8	91.8	65.2	120.4
YOLOv9m [46]	One-Stage	77.0	20.1	94.8	89.2	91.5	64.8	103.1
YOLOv10m [47]	One-Stage	58.9	15.3	93.6	87.5	89.2	61.2	105.2
YOLOv11m [48]	One-Stage	58.9	15.3	93.8	87.8	89.5	61.5	116.5
Faster R-CNN [15]	Two-Stage	213.3	41.3	95.6	90.3	92.2	66.8	51.2
DINO Deformable-DETR [23]	DETR	279.0	47.5	96.2	91.2	93.5	68.2	27.4
DEIM-D-Fine-M [49]	DETR	56.37	19.19	95.4	89.2	92.8	65.8	69.9
D-Fine-M [50]	DETR	56.37	19.19	95.2	88.9	91.5	65.5	70.2
RT-DETR-L [21]	DETR	103.4	31.9	95.9	90.7	92.3	67.5	60.1
RT-DETR-r50 [21]	DETR	129.6	41.9	96.1	91.1	92.4	67.8	58.1
RT-DETR-r34 [21]	DETR	88.8	31.1	95.4	90.1	92.0	66.5	62.3
RT-DETR-r18 [21]	DETR	56.9	19.87	95.8	90.9	93.5	66.0	78.6
HSF-DETR (ours)	DETR	59.7	18.07	97.4	95.1	96.6	70.6	70.2

**Table 6:** Cross-domain generalization results on VisDrone2019 and BDD100K datasets.

Dataset	Model	P (%)	R (%)	mAP50 (%)	mAP50-95 (%)
Visdrone2019	RT-DETR-R18	61.0	46.6	47.4	29.2
	HSF-DETR	62.9	47.1	48.3	29.0
BDD100K	RT-DETR-R18	64.2	47.2	49.1	31.9
	HSF-DETR	65.0	47.9	50.3	32.8

## Data Availability

The data presented in this study are available upon request from the corresponding author.
